# Small Population Size and Low Levels of Genetic Diversity in an Endangered Species Endemic to the Western Tianshan Mountains

**DOI:** 10.3390/plants14193105

**Published:** 2025-10-09

**Authors:** Zhihao Su, Zhiye Lin, Li Zhuo, Xiaolong Jiang, Qichuan Jiang

**Affiliations:** 1 Xinjiang Key Laboratory of Special Species Conservation and Regulatory Biology, College of Life Science, Xinjiang Normal University, Urumqi 830017, China; 2 Library, Xinjiang Normal University, Urumqi 830017, China; 3 The Laboratory of Forestry Genetics, Central South University of Forestry and Technology, Changsha 410004, China; 4 Yunan Key Laboratory of Forest Plant Cultivation and Utilization, Yunnan Academy of Forestry & Grassland, Kunming 650204, China

**Keywords:** *Ammopiptanthus nanus*, SNP, genetic diversity, genetic structure, gene flow

## Abstract

*Ammopiptanthus nanus* is an endangered evergreen shrub endemic to the western Tianshan Mountains. Genetic diversity and population structure of this species were assessed using single-nucleotide polymorphism (SNP) loci identified via double-digest restriction site-associated DNA (ddRAD) sequencing. In this study, a total of 42 individuals were sampled from seven populations located in valley habitats across the western Tianshan Mountains. A low level of genetic diversity (mean *H*_E_ = 0.09) and strong interpopulation genetic differentiation (mean *F*_ST_ = 0.4832) were observed in the species, indicating substantial genetic structuring among populations. Population structure analyses using Admixture analysis, principal coordinate analysis (PCA), and maximum likelihood trees yielded congruent patterns, supporting four genetically distinct groups within the western Tianshan Mountains. Genetic drift and inbreeding, likely induced by habitat fragmentation, appear to be primarily responsible for the low genetic diversity, while restricted gene flow probably shaped the pronounced genetic structure. Based on our findings, we recommend specific conservation strategies for *A. nanus* aimed at maintaining genetic diversity and facilitating gene flow.

## 1. Introduction

Habitat fragmentation is the process by which a large, continuous habitat is broken up into smaller, discontinuous patches, either by human activity or natural causes [1,2]. Habitat fragmentation theoretically has negative genetic effects, in the form of increased random genetic drift and allele fixation, and the consequent erosion of genetic diversity, through population size reduction and increased spatial isolation [3,4]. Along with the increased spatial isolation of populations, gene flow is susceptible to being impeded [5,6]. A lack of gene flow increases the chance of inbreeding in isolated habitat remnants, thereby decreasing heterozygosity and evolutionary potential [4]. Increased random genetic drift and elevated inbreeding trap threatened plants in extinction vortices, greatly increasing extinction risk [6]. However, the genetic response to habitat fragmentation varies among threatened plants, as some factors can buffer against the adverse effects associated with fragmentation [5].

Long-lived plants with extended generation times often exhibit delayed responses to environmental changes, a phenomenon known as ecological hysteresis. This delay can make them more resistant to the immediate effects of genetic drift, as genetic changes accumulate more slowly over multiple generations [5,7]. Moreover, short-term habitat fragmentation may not cause significant genetic changes in populations, as genetic drift and inbreeding require multiple generations to act on populations [8]. Furthermore, if the species were capable of producing abundant pollen and seeds, and of long-distance dispersal by wind and animal transport, high gene flow between populations would connect small patches into metapopulations, ultimately increasing the effective population size [2,5].

*Ammopiptanthus* is an evergreen broad-leaved shrub, a relic plant of Tertiary origin, occupying mainly sand dunes, gravel slopes, and terraces next to ravines in Central Asia [9,10]. The genus consists of only two species, *A. mongolicus* and *A. nanus*. *Ammopiptanthus mongolicus* is primarily distributed in the Alxa–Ordos region, extending north to southern Mongolia, and *A. nanus* is endemic to the western Tianshan Mountains, occupying valleys in Wuqia County, China, and Naryn State, Kyrgyzstan [11]. The species has been listed as endangered on the Red List of Threatened Species in China due to continuous demographic decline over the past few decades [12].

Mapping the genetic diversity within an endangered species allows managers to optimize conservation strategies, and is increasingly being used for conservation interventions [13]. The genetic diversity and population structure of *A. nanus* have been examined using several first-generation molecular markers to date, but inconsistent conclusions exist. A low level of genetic diversity and strong genetic differentiation between populations were detected using AFLP or chloroplast intergenic spacers [14,15], while low levels of genetic differentiation between populations were inferred using ETS-SSR and ISSR markers [16,17]. With the development of second-generation sequencing techniques, the population genetics of this species should be reassessed using techniques that generate more polymorphic loci.

Single-nucleotide polymorphisms (SNPs) have been widely used in population genetics studies due to their high polymorphism, lack of ascertainment bias, and wide genomic distribution [18]. Several types of high-throughput methods have been developed to discover polymorphic SNP loci, one of which is double-digest restriction site-associated DNA (ddRAD) sequencing. This method can produce high-density genome-wide SNPs, does not require a reference genome sequence, and can estimate genetic diversity with high efficiency [19]. We selected this method to obtain genome-level variants for *A. nanus*, aiming to address the following questions in this study: (i) Has the genetic diversity of *A. nanus* been eroded due to long-term habitat fragmentation? (ii) Do the remnant populations exhibit strong interpopulation genetic differentiation?

## 2. Results

### 2.1. SNPS from RAD-seq Analysis of A. nanus

The average number of raw reads per sample was 9,303,109, with a range of 6,463,834 to 15,707,966. After filtering out low-quality reads, the average number of raw reads per sample was 8,805,891, with a range of 6,161,376 to 14,953,318. The average depth of the samples was 17.20, with a range of 12.73 to 22.31, and the average number of tags per sample was 455,454, with a range of 328,390 to 842,442. After filtering under optimal conditions, a total of 83,735 SNPs were detected on average.

### 2.2. Genetic Diversity of A. nanus

**Figure 1:**
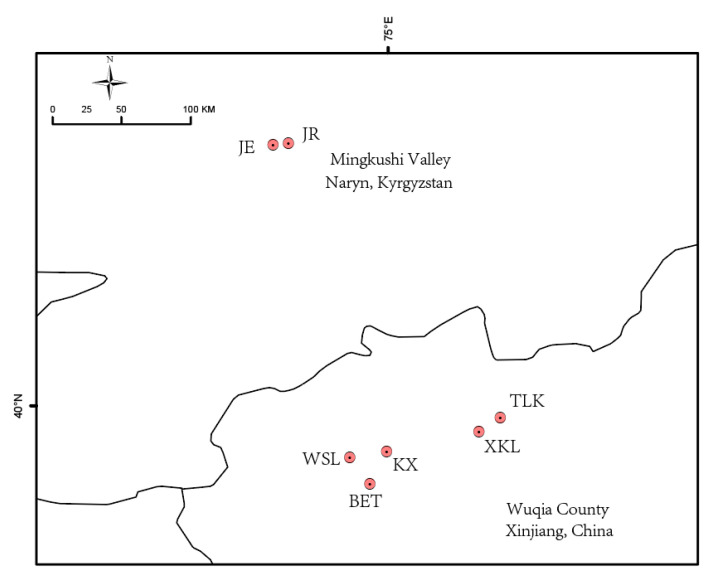
Geographic location of *A. nanus* populations in the western Tianshan Mountains. Population IDs correspond to those in Table 1.

The geographic locations of *A. nanus* populations are shown in Figure 1. The genetic diversity indexes are presented in Table 1. Positive *F*_IS_ values were found in the populations KX, BET, and XKL, suggesting a lack of heterozygotes and inbreeding in these locations, whereas negative *F*_IS_ values were found in populations JR, JE, and TLK, suggesting an excess of heterozygotes within them [20]. Wright (1965) categorized the genetic differentiation between populations as “large” when *F*_ST_ > 0.25, “significant” when 0.15 < *F*_ST_ ≤ 0.25, “moderate” when 0.05 ≤ *F*_ST_ ≤ 0.15), or “not different” when *F*_ST_ < 0.05 [21]. The mean genetic differentiation between the populations (*F*_ST_) was 0.4832, ranging from 0.1579 to 0.7339 (Table 2). Thus, *A*. *nanus* exhibited significant genetic differentiation among populations.

### 2.3. Genetic Structure of A. nanus

**Figure 2:**
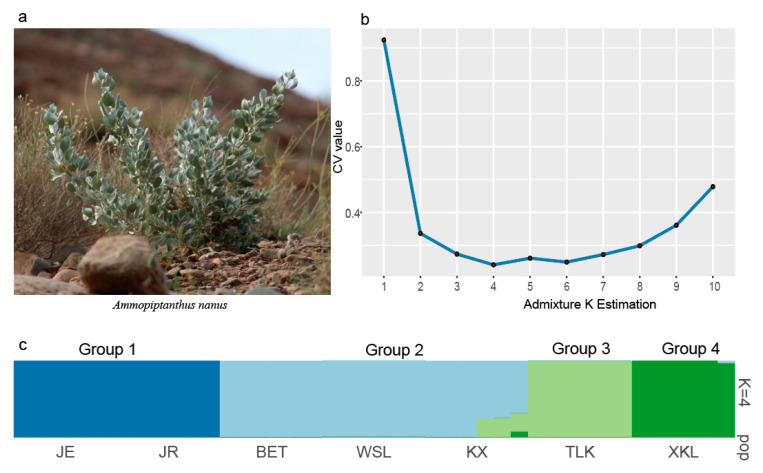
(**a**) Individual of *A. nanus*. (**b**) A barplot of individuals from 7 *A. nanus* populations using the program Admixture. Four inferred groups are represented by four colors (light green, green, light blue, and blue). Each bar represents an individual with assignment probabilities to each group. The labels below the barplot refer to the site code in Table 1. The labels above the barplot represent how the sampled populations are associated with the inferred groups. (**c**) The mean cross-validation (CV) error for genetically distinct groups (K, from K = 2 to 10).

**Figure 3:**
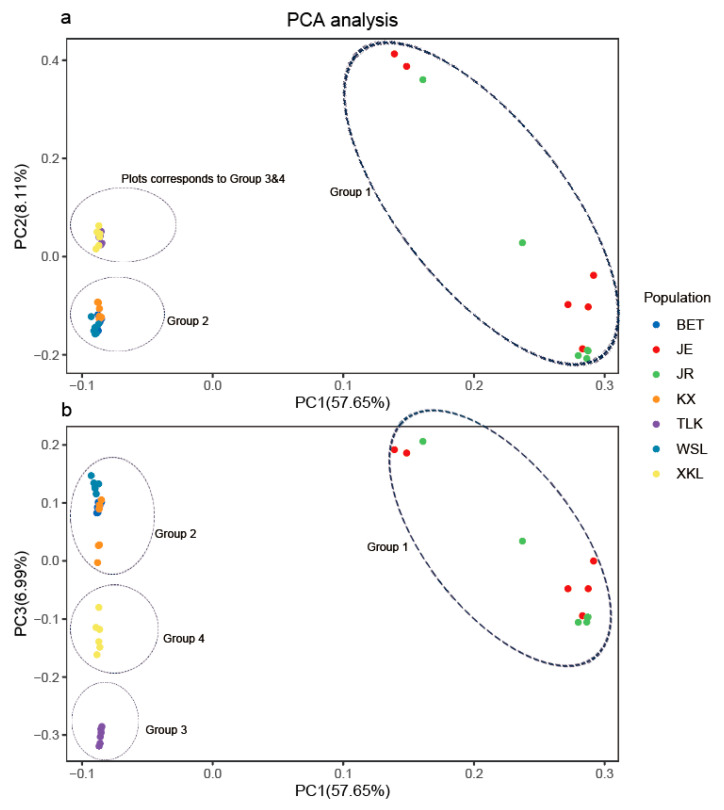
The principal component analysis (PCA) based on the SNP data. PC1, PC2, and PC3 explained 57.65, 8.11, and 6.99% of the variation, respectively. (**a**) PCA based on PC1 and PC2; (**b**) PCA based on PC1 and PC3.

**Figure 4:**
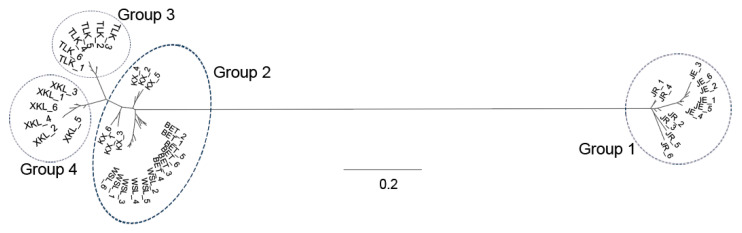
Maximum likelihood (ML) phylogenetic trees for 42 *A. nanus* individuals. Branches were circled corresponding to the grouping pattern in the Admixture analysis.

**Figure 5:**
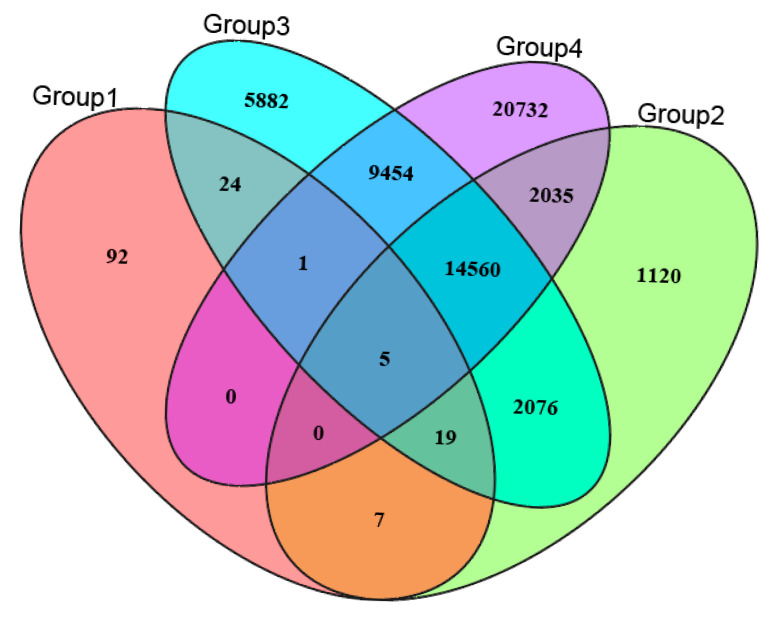
SNP sharing between genetic groups of *A. nanus*.

**Figure 6:**
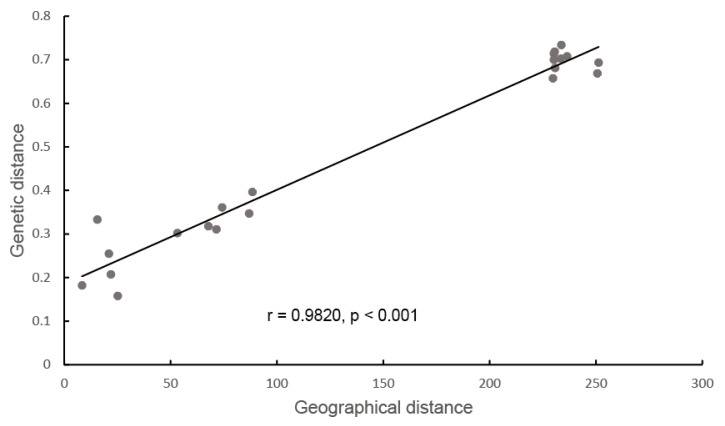
The linear relationship between geographic and genetic distance (r = 0.9820, *p* < 0.001).

**Figure 7:**
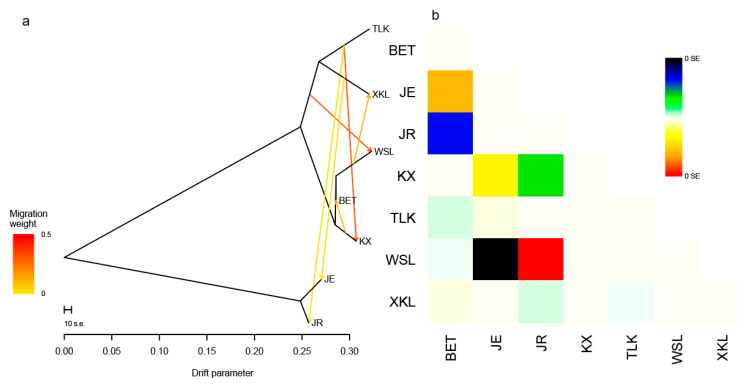
(**a**) Maximum likelihood (ML) tree of seven populations of *A. nanus* with inferred migration edges. Migration events are represented by color-scaled arrows, with gradient intensity corresponding to relative migration weight. (**b**) Residual fit plotted of seven *A. nanus* populations. Positive residual value indicates higher genetic relatedness.

The lowest CV error value showed by the Admixture analysis was 0.2405 for K = 4; therefore, K = 4 was chosen as the optimal genetic grouping pattern (Figure 2b). The populations JE and JR were assigned to Group 1; KX, BET, and WSL to Group 2; TLK to Group 3; and XKL to Group 4 (Figure 2c). In the PCA diagram, the first, second, and third axes accounted for 57.65%, 8.11%, and 6.99% of the total variation, respectively (Figure 3a,b). The grouping patterns shown by PC 1 and 2 were partially disentangled from the Admixture assignments, where the plots of populations KX and TLK were mixed together, while the patterns showed by PC 2 and 3 were fully consistent with the Admixture assignments (Figure 3b). In the ML tree, individuals were also clustered into four distinct groups, the same pattern as that in the Admixture assignments (Figure 4). The SNP sharing analysis showed that Group 1 shared a small number of SNPs with Groups 2, 3, and 4, with values of 31, 49, and 6, respectively; Group 2 shared a large number of SNPs with Group 3 and 4, with values of 16,660 and 16,600, respectively; and Group 3 shared the largest number of SNPs (24,020) with Group 4 (Figure 5). The results of the SNP sharing analysis were consistent with the *F*_ST_ values between the populations (Table 2); low genetic differentiation between populations with low *F*_ST_ values also indicated a large number of shared SNPs. AMOVA results showed that 93.99% (*p* < 0.0001) of the total variation occurred among populations, and only 6.01% occurred within populations (Table 3). For the Mantel test of the *A. nanus* populations, the genetic distance was significantly linearly correlated with geographical distance with a positive coefficient (Figure 6, r = 0.9820, *p* < 0.001). For the Treemix analysis, the optimal migration parameter was set to m = 6 based on the minimization of residuals. The Treemix phylogenetic network topology was consistent with the ML phylogeny, with populations JE and JR forming a monophyletic clade, while the remaining five populations formed a sister clade (Figure 7). Significant asymmetric gene flow was detected from population TLK to KX, and from TLK-XKL to WSL.

## 3. Discussion

### 3.1. Genetic Diversity of A. nanus

Expected heterozygosity (*H*_E_) is a critical index used to detect the level of genetic diversity of a population [22]. Generally, the genetic diversity level of species with a narrow range is lower than that of species with a wider range [23]. When compared to another species within the same genus, *Ammopiptanthus mongolicus*, which has a more widespread distribution in the Alxa–Ordos region of northwestern China (ddRAD, *H*_E_ = 0.17, unpublished), *A. nanus* exhibits a rather low level of genetic diversity (*H*_E_ = 0.09). *Ammopiptanthus mongolicus* has a larger population size than *A. nanus*, suggesting that *A. nanus*’s genetic diversity is more impacted by genetic drift than in *A. mongolicus*. This value is also lower than that of another species of Fabaceae widespread in the same region, *Medicago falcata* L. (SLAF-seq, *H*_E_ = 0.22) [24], and much lower than that of another two widespread species in the Fabaceae, Soybean (*Glycine max* L. Merrill) (DArT-seq, *H*_E_ = 0.39) and Cowpea (*Vigna unguiculata* L. Walp) (KASP-SNP, *H*_E_ = 0.37) [22,25]. The widespread distribution of plants is associated with increased opportunities for outcrossing, which provides a beneficial effect in maintaining genetic diversity [26]. While habitat fragmentation reduces size and increases spatial isolation of populations, such changes may lead to an erosion of genetic variation through increased random genetic drift and elevated inbreeding [27].

Genetic diversity provides the basis for evolutionary potential, which is necessary for the survival and adaptability of a species. A lower genetic diversity may indicate that a species is less likely to adapt to changes in its environment [28]. The observed low level of genetic diversity of *A. nanus* is likely primarily due to the increased genetic drift and inbreeding associated with habitat fragmentation. The high level of genetic differentiation between *A. nanus* populations suggests that genetic drift is dominant within the species (Table 2). As a relict of Tertiary origin, *A. nanus* occupies scattered valleys in the western Tianshan Mountains [14,29]. The populations are generally geologically isolated from each other and have a rather small population size. Small isolated populations are subjected to increased random genetic drift, leading to a loss of genetic variation [17,30]. Meanwhile, inbreeding may also facilitate the erosion of genetic variation within *A. nanus*. Three of the seven sampled populations, namely KX, BET, and XKL, showed positive *F*_IS_ values, suggesting the existence of inbreeding within these populations [20]. *Ammopiptanthus nanus* comprises both self-incompatible and outcrossed breeding systems [14]. During the flower season, *A. nanus* displays an explosive flowering pattern, which results in hundreds of hermaphrodite flowers opening at the same time, easily attracting pollinators to visit the flowers of the same plant [14,31]. As a result, the chance of inbreeding increases, leading to a reduction in heterozygosity at the locus [32].

The erosion of genetic diversity resulting from habitat fragmentation, as found in *A. nanus*, would reduce population fitness and viability and limit the ability to respond to changing environmental pressures, involving the species in an extinction vortex [27].

### 3.2. Genetic Structure of A. nanus

The SNP sharing analysis, the mean *F*_ST_ value (0.4832), and the AMOVA indicated a high level of genetic differentiation between *A. nanus* populations (Figure 5; Table 2 and Table 3). The Admixture analysis, PCA, and ML tree all divided the seven populations of *A. nanus* into four genetically differentiated groups (Figure 2, Figure 3 and Figure 4), indicating that habitat fragmentation has profoundly shaped the distribution patterns of genetic variation in *A. nanus*.

Generally, the level of gene flow between populations is negatively correlated with the degree of genetic differentiation between them [33]. Most genetic differentiation between populations exceeded 0.25, indicating a strong population structure in *A. nanus*. The pronounced structure was further supported by restricted asymmetric gene flow between some populations (e.g., KX→BET, TLK→JR, WSL→XKL, and XKL→JE) in the Treemix analysis (Figure 7). Together, these findings indicate a strong population structure in *A. nanus*. *Ammopiptanthus nanus* is pollinated by insects, with the main pollinators being *Anthophora* (*Dasymegilla*) *waltoni* Cockerell, *Megachile* (*Chalcodoma*) sp., and *Halicls* sp. These insects fly a short distance, generally <2.5 km, substantially less than the geographical distance between the populations [31]. Consequently, pollinator scarcity and short flight distances may result in low flower visit frequencies, and thus reduce pollination efficiency [2]. Moreover, the seeds of *A. nanus* are large and heavy, like those of *A. mongolicus*, so can only disperse across a short distance by gravity [14,34]. Thus, the short dispersal of propagules may greatly restrict interpopulation gene flow, increasing the chances of random genetic drift and inbreeding over generations, consequently elevating the genetic differentiation [35]. Even with the existing interpopulation genetic divergence, the population KX, which was assigned to Group 2 and adjacent to TLK (Group 3), showed a genetic mixture of the two groups. The intersection of gene flow at KX here was primarily due to spatial proximity to the neighboring populations, BET, WSL, and TLK.

The Mantel test showed a strong positive correlation between the genetic distances and the geographic distances (Figure 6, r = 0.9820, *p* < 0.001), suggesting an increased genetic heterogeneity between the populations accompanying increasing geographic distances. *Ammopiptanthus nanus* populations are scattered in the deep valleys, almost entirely isolated from each other. The high mountains, acting as natural barriers, represent another great constraint on the gene flow. Other shrubs occupying valleys of the Tianshan Mountains, such as *Heliantemum songaricum*, also show a positive correlation between genetic and geographical distance [36].

### 3.3. Conservation Implications

Associated with habitat fragmentation, the low level of genetic diversity, weak interpopulation gene flow, and clear interpopulation divergence found in *A. nanus* warrant conservation attention to promote adaptability. Given the small remnant population sizes, we consider that the first urgent measure is to protect all the individuals to ensure their sustainable survival at their original sites. A nature reserve should be established in Wuqia County to decrease the impact of human activities and promote the natural regeneration of *A. nanus*. Information should be disseminated to local herders with the aim of minimizing firewood gathering and grazing. Also, fences should be built around the populations to prevent livestock accessing the land. In addition, we recommend supplementary hand-pollination outcrossing from nearby populations within the genetic group during the flower season to reduce inbreeding depression and enhance reproductive fitness. However, relying solely on in situ conservation would be one-sided and finally not realistic. Therefore, we recommend implementation of ex situ conservation, which is also an extremely demanding conservation strategy for endangered species [37]. Ex situ conservation is generally associated with botanical gardens [38], which store germplasm with maximal genetic representation [39]. We recommend the establishment of botanical gardens, implementing the following strategies: (i) collect seeds from as many individuals as possible from all *A. nanus* groups; (ii) propagate seedlings in different colonies according to the subdivided groups; and (iii) carry out garden trials to measure fitness in outcrossing progeny between different groups, assessing the risks of outbreeding depression in a controlled setting, and validate the benefits of introducing novel genotypes between groups [40]. The low levels of gene flow found in *A*. *nanus* would diminish its long-term viability, and thus the augmentation of gene flow across populations is critical for maintaining viability. As seedlings are propagated, re-establishing populations between existing sites to enhance connectivity is urgently recommended.

## 4. Materials and Methods

### 4.1. Plant Materials

*Ammopiptanthus nanus* is typically 40–70 cm tall and grows on gravel slopes and terraces alongside ravines [9,11]. The stems are terete, weakly ridged, and, at first, gray puberulent, but glabrescent when grown. The leaves are silvery tomentose, with 1 or 3 folioles. There are usually 4–15 flowers, in short dense terminal racemes, and the legumes are linear, with 2–4 seeds. The species excels in drought, cold, and salty conditions, making it widely used in desertification control, and owing to its evergreen character, it is commonly used for garden ornamental purposes [29,41].

*Ammopiptanthus nanus* is scattered in valleys of the western Tianshan Mountains. The valleys are separated from each other by long distances, so each geographic location was treated as a population. A total of 42 individuals were sampled from 7 populations of the species, 5 in valleys of Wuqia County in China (WSL, KX, BET, TLK, XKL) and 2 in the Mingkushi Valleys in Kyrgyzstan (JR, JE). Six individuals were randomly sampled from each population, recording latitude, longitude, and altitude (Table 1; Figure 1). This sample size is limited but reflects small population size. The identification of the species was performed by Li Zhuo, and voucher herbarium specimens were stored at Xinjiang Normal University. Fresh leaves from each individual were placed in self-seal bags containing silica gel to ensure rapid dehydration and drying, and stored at 4 °C for DNA extraction.

### 4.2. DNA Extraction and RAD Sequencing

Total genomic DNA was extracted from dried leaf tissues with a modified 2×CTAB method [42], then purified by precipitation of polysaccharides [43]. Two high-fidelity restriction enzymes, HindIII and Bfal, were selected to prepare the RAD-seq library [19]. For each individual, digestion was performed in 30 μL reactions, followed by ligation of the P1 and P2 adapters. The DNA library was purified and amplified by PCR, followed by agarose gel purification (2%) and selection of sizes between 220 and 450 bp. All 42 samples were sequenced on an Illumina NovaSeq (2×150, Illumina, San Diego, CA, USA). The creation of the RAD library and sequencing was performed by Personalbio, Inc. (Shanghai, China).

### 4.3. SNP Calling

Raw reads were filtered with Stacks v.2.55 to obtain high-quality reads, ensuring sequence assembly accuracy [44]. First, reads containing 3’-end contaminants were trimmed, followed by quality filtering with Trimmomatic v.0.39 using a sliding window approach based on FastQC reports (http://www.bioinformatics.babraham.ac.uk/projects/fastqc, accessed on 10 November 2024). Quality control parameters included sliding-window trimming (window size: 5 bp; step size: 1 bp), removing regions with an average Phred score < 30, and discarding reads containing Ns or with post-trimming lengths < 50 bp [45,46].

For *A. nanus*, which belongs to a monophyletic genus, no phylogenetically close reference genome was available. Reads were processed de novo to generate consensus loci, with the individual showing the highest locus count selected as the pseudo-reference. Clean reads were mapped to this pseudo-reference using Bowtie2 v.2.4.5 [47]. We optimized stacks parameters to maximize polymorphic loci (*π* > 0.01) while minimizing false positives (FPR < 5%) using simulated RAD-seq data [48]: the minimum number of perfectly matching raw reads required to create stacks (m: 2–6); the maximum nucleotide distance allowed to merge stacks within an individual (M: 2–12); the maximum number of mismatches to merge stacks across individuals (n: 1–6); the minimum minor allele frequency (min_maf = 0.05); the minimum percentage of individuals across populations required to process a locus (r = 0.5); and the minimum occurrence frequency of a locus (*p* = 0.5). Parameters were individually perturbed with others fixed. The optimal combination (m = 3, M = 3, n = 4) was determined by log-likelihood scoring [49]. To reduce the effect of linkage disequilibrium on genetic structure, only one SNP was retained for each contig of the pseudo-reference genome.

### 4.4. Genetic Diversity and Structure

Four indices of genetic diversity, nucleotide diversity (*π*), expected heterozygosity (*H*_E_), observed heterozygosity (*H*o), and inbreeding coefficient (*F*_IS_), were estimated for each *A. nanus* population using Stacks v.2.55 [44]. Pairwise *F*_ST_ values between populations, representing the genetic distance, were also calculated with this software.

Genetic structure was inferred through maximum likelihood estimation implemented in Admixture v.1.30 [50], under the assumption of linkage equilibrium among all analyzed loci. We also assessed genetic structure using principal component analysis (PCA), maximum likelihood (ML) trees, and analysis of molecular variance (AMOVA). To obtain the optimal number of genetically distinct groups, the mean cross-validation (CV) error for each K was calculated using ten replicates (from K = 2 to 10). The optimal K was determined by the lowest mean CV value. Each run was conducted with 200,000 Markov Chain Monte Carlo (MCMC) generations and a burn-in period of 100,000 generations. The PCA was conducted using the glPCA function in the “adegenet” package, implemented in R v.3.2.3 [51]. To demonstrate the genetic patterns distributed in geographical space, the first three components of the PCA were calculated using the Procrustes method in the “vegan” package in R [52]. The ML tree was constructed in RAxML v.8.2.4 using the GTR+GAMMA model with 1000 non-parametric bootstrap replicates [53]. After converting the SNP data into the arp format, AMOVA was performed in ARLEQUIN v.3.01 [54]. After identifying genetic groups, SNP sharing across groups was analyzed using a custom script, and the diagram was created with the VennDiagram package in R [55].

To examine geographical distance effects on the genetic structure, we performed a Mantel test in ARLEQUIN v.3.01 [54]. Geographic distances between populations were calculated using GEODIS v.2.5 [56], while genetic distances between populations were represented by previously calculated pairwise *F*_ST_ values. We tested gene flow using Treemix v1.13’s composite-likelihood approach [57]. Phylogenetic networks were reconstructed with an increasing number of migration edges* (m = 0–10). Model selection was based on residual analysis, with the optimal migration parameter determined by minimizing model residuals.

## 5. Conclusions

With the SNPs obtained from ddRAD sequencing, a low level of genetic diversity and strong interpopulation differentiation were found within *A. nanus*. The increased genetic drift and inbreeding resulting from habitat fragmentation were speculated to be primarily associated with the low genetic diversity, and the low level of gene flow between the isolated populations was suggested to have shaped the strong genetic structure. To sum up, habitat fragmentation has deeply affected the population genetics of *A. nanus*. Our findings provide useful conservation implications for *A. nanus*. Together with in situ conservation, we call for the propagation of seedlings of *A. nanus* in botanical gardens to re-establish populations between the existing sampled sites to strengthen their genetic links. Despite the polymorphism of SNP loci obtained using ddRAD sequencing, the coverage of the whole genome and the depth of SNP data analysis can be greatly improved with whole-genome resequencing in future population genetics studies of *A*. *nanus*.

## Figures and Tables

**Table 1:** Sampling information and genetic diversity of *A. nanus* populations based on SNP data. *H*_O_ observed heterozygosity; *H*_E_ expected heterozygosity; *π*: mean nucleotide diversity; *F*_IS_: inbreeding level.

Population ID	Latitude	Longitude	Altitude (m)	Sample	*H*o	*H*_E_	*π*	*F*_IS_
JR	74.35	41.71	1730	6	0.12	0.09	0.11	−0.01
JE	74.25	41.70	1693	6	0.11	0.09	0.10	−0.01
WSL	74.75	39.66	2290	6	0.09	0.08	0.09	0.00
KX	74.99	39.70	2167	6	0.10	0.11	0.13	0.05
BET	74.88	39.49	2512	6	0.11	0.10	0.11	0.01
TLK	75.73	39.92	2212	6	0.10	0.08	0.09	−0.01
XKL	75.59	39.83	2109	6	0.10	0.09	0.10	0.01

**Table 2:** Pairwise estimated values of *F*_ST_ (up diagonal) between the populations of *A. nanus*.

Population	JR	JE	WSL	KX	BET	TLK	XKL
JR		0.1822	0.7002	0.6575	0.6687	0.7138	0.7024
JE			0.7187	0.6810	0.6933	0.7339	0.7078
WSL				0.2550	0.2073	0.3966	0.3610
KX					0.1579	0.3177	0.3020
BET						0.3469	0.3107
TLK							0.3332
XKL							

**Table 3:** Results of the analysis of molecular variance for 7 populations of *A. nanus* based on RAD-seq SNP data.

Source of Variation	d.f.	Sum of Squares	Variance Components	Percentage of Variation
Among populations	6	351,886.83	9671.65	93.99 *
Within populations	35	21,627.00	617.91	6.01 *
Total	41	373,513.84	10,289.56	

* *p* < 0.0001.

## Data Availability

Illumina sequence read data obtained in this study are available through NCBI BioProject PRJNA1108609.

## References

[1] Ellwanger C., Steger L., Pollack C., Wells R., Benjamin Fant J. (2022). Anthropogenic fragmentation increases risk of genetic decline in the threatened orchid *Platanthera leucophaea*. Ecol. Evol..

[2] Aguilar R., Cristóbal-Pérez E.J., Balvino-Olvera F.J., de Jesús Aguilar-Aguilar M., Aguirre-Acosta N., Ashworth L., Lobo J.A., Martén-Rodríguez S., Fuchs E.J., Sanchez-Montoya G. (2019). Habitat fragmentation reduces plant progeny quality: A global synthesis. Ecol. Lett..

[3] Soons M.B., Messelink J.H., Jongejans E., Heil G.W. (2005). Habitat fragmentation reduces grassland connectivity for both short-distance and long-distance wind-dispersed forbs. J. Ecol..

[4] Schlaepfer D.R., Braschler B., Rusterholz H.-P., Baur B. (2018). Genetic effects of anthropogenic habitat fragmentation on remnant animal and plant populations: A meta-analysis. Ecosphere.

[5] Chen X.Y. (2000). Effects of habitat fragmentation on genetic structure of plant populations and implications for the biodiversity conservation. Acta Ecol. Sin..

[6] Honnay O., Jacquemyn H. (2007). Susceptibility of common and rare plant species to the genetic consequences of habitat fragmentation. Conserv. Biol..

[7] Morden C.W., Loeffler W. (1999). Fragmentation and genetic differentiation among subpopulations of the endangered Hawaiian mint *Haplostachys haplostachya* (Lamiaceae). Mol. Ecol..

[8] Evans M.J., Banks S.C., Driscoll D.A., Hicks A.J., Melbourne B.A., Davies K.F. (2017). Short- and long-term effects of habitat fragmentation differ but are predicted by response to the matrix. Ecology.

[9] Cui H.B., Wu Z.Y., Raven P.H. (1998). Fabaceae (5). Flora of China.

[10] Cao S., Wang Y., Li X., Gao F., Feng J., Zhou Y. (2020). Characterization of the AP2/ERF transcription factor family and expression profiling of DREB subfamily under cold and osmotic stresses in *Ammopiptanthus nanus*. Plants.

[11] Shen G.M., Shen G.M., Mao Z.M. (2011). Fabaceae-Apiaceae. Flora Xinjiangensis.

[12] Fu G.L. (1991). Rare and Endangered Plants in China.

[13] Brooks E., Slender A.L., Cu S., Breed M.F., Stangoulis J.C. (2022). A range-wide analysis of population structure and genomic variation within the critically endangered spiny daisy (*Acanthocladium dockeri*). Conserv. Genet..

[14] Chen G.-Q., Huang H.-W., Crawford D.J., Pan B.-R., Ge X.-J. (2009). Mating system and genetic diversity of a rare desert legume *Ammopiptanthus nanus* (Leguminosae). J. Syst. Evol..

[15] Su Z., Richardson B.A., Zhuo L., Jiang X., Li W., Kang X. (2017). Genetic diversity and structure of an endangered desert shrub and the implications for conservation. AoB Plants.

[16] Liu J., Wang X., Lu T., Wang J., Shi W. (2023). Identification of the efficacy of ex situ conservation of *Ammopiptanthus nanus* based on its ETS-SSR markers. Plants.

[17] Ge X.-J., Yu Y., Yuan Y.-M., Huang H.-W., Yan C. (2005). Genetic diversity and geographic differentiation in endangered *Ammopiptanthus* (Leguminosae) populations in desert regions of northwest China as revealed by ISSR analysis. Ann. Bot..

[18] Basak M., Uzun B., Yol E. (2019). Genetic diversity and population structure of the Mediterranean sesame core collection with use of genome-wide SNPs developed by double digest RAD-Seq. PLoS ONE.

[19] Peterson B.K., Weber J.N., Kay E.H., Fisher H.S., Hoekstra H.E. (2012). Double digest RADseq: An inexpensive method for de novo SNP discovery and genotyping in model and non-model species. PLoS ONE.

[20] Weir B.S., Cockerham C.C. (1984). Estimating F-statistics for the analysis of population structure. Evol. Int. J. Org. Evol..

[21] Wright S. (1965). The interpretation of population structure by F-statistics with special regard to systems of mating. Evolution.

[22] Potts J., Michael V.N., Meru G., Wu X., Blair M.W. (2024). Dissecting the genetic diversity of USDA cowpea germplasm collection using kompetitive allele specific PCR-single nucleotide polymorphism markers. Genes.

[23] Atasagun B. (2022). Assessment of the genetic diversity of a critically endangered species *Centaurea amaena* (Asteraceae). Arch. Biol. Sci..

[24] Du Y., Yu X.M., Wang P., Li Q., Wang Y.X., Zhang B. (2024). Genetic diversity analysis of wild *Medicago falcata* L. in Xinjiang based on SNP molecular markers. Feed Res..

[25] Shaibu A.S., Ibrahim H., Miko Z.L., Mohammed I.B., Mohammed S.G., Yusuf H.L., Kamara A.Y., Omoigui L.O., Karikari B. (2021). Assessment of the genetic structure and diversity of Soybean (*Glycine max* L.) germplasm using diversity array technology and single nucleotide polymorphism markers. Plants.

[26] Frankham R. (1995). Conservation genetics. Ann. Rev. Genet..

[27] Young A., Boyle T., Brown T. (1996). The population genetic consequences of habitat fragmentation for plants. Trends Ecol. Evol..

[28] Li S., Gan X., Han H., Zhang X., Tian Z. (2018). Low within-population genetic diversity and high genetic differentiation among populations of the endangered plant *Tetracentron sinense* Oliver revealed by inter-simple sequence repeat analysis. Ann. For. Sci..

[29] Du Z., He Y., Wang H., Wang C., Duan Y. (2021). Potential geographical distribution and habitat shift of the genus *Ammopiptanthus* in China under current and future climate change based on the MaxEnt model. J. Arid Environ..

[30] Anto M., Anjala M., Jothish P.S., Rameshkumar K.B., Padmesh P., Anilkumar C. (2020). Population genetic structure of *Garcinia imberti* Bourd. an endangered endemic tree of southern Western Ghats, India. Plant Sci. Today.

[31] Li A., Ma M., Li H., He S., Wang S. (2023). Genetic diversity and population differentiation of a Chinese endangered plant *Ammopiptanthus nanus* (M. Pop.) Cheng f. Genes.

[32] Chávez-Cortázar A., Oyama K., Ochoa-Zavala M., Mata-Rosas M., Veltjen E., Samain M.S., Quesada M. (2021). Conservation genetics of relict tropical species of *Magnolia* (section *Macrophylla*). Conserv. Genet..

[33] Cai C., Xiao J., Ci X., Conran J.G., Li J. (2021). Genetic diversity of *Horsfieldia tetratepala* (Myristicaceae), an endangered plant species with extremely small populations to China: Implications for its conservation. Plant Syst. Evol..

[34] Shi W., Su Z.H., Liu P.L., Pan B.R., Zhao Y.F., Wang J.C. (2017). Molecular, Karyotypic, and Morphological evidence for *Ammopiptanthus* (Fabaceae) taxonomy. Ann. Mo. Bot. Gard..

[35] Cristóbal-Pérez E.J., Fuchs E.J., Martén-Rodríguez S., Quesada M. (2021). Habitat fragmentation negatively affects effective gene flow via pollen, and male and female fitness in the dioecious tree, *Spondias purpurea* (Anacardiaceae). Biol. Conserv..

[36] Zhuo L., Su Z., Zhao H., Jiang X., Zhang L. (2024). Genetic structure of two endangered shrubs in Central Asia and northwestern China and the implications for conservation. Plant Syst. Evol..

[37] Kashimshetty Y., Pelikan S., Rogstad S.H. (2017). Effective seed harvesting strategies for the ex situ genetic diversity conservation of rare tropical tree populations. Biodivers. Conserv..

[38] Chen G., Sun W. (2018). The role of botanical gardens in scientific research, conservation, and citizen science. Plant Divers..

[39] Blackmore S., Gibby M., Rae D. (2011). Strengthening the scientific contribution of botanic gardens to the second phase of the Global Strategy for Plant Conservation. Bot. J. Linn. Soc..

[40] Ottewell K.M., Bickerton D.C., Byrne M., Lowe A.J. (2016). Bridging the gap: A genetic assessment framework for population-level threatened plant conservation prioritization and decision-making. Divers. Distrib..

[41] Wei H., Wu P., Ge X., Liu M., Wei X. (2007). Chemical constituents of the seeds of *Ammopiptanthus* (Leguminosae) and their systematic and ecological significance. Biochem. Syst. Ecol..

[42] Doyle J.J., Doyle J.L. (1987). A rapid DNA isolation procedure for small quantities of fresh leaf tissue. Phytochem. Bull..

[43] Michaels S.D., John M.C., Amasino R.M. (1994). Removal of polysaccharides from plant DNA by ethanol precipitation. BioTechniques.

[44] Catchen J., Hohenlohe P.A., Bassham S., Amores A., Cresko W.A. (2013). Stacks: An analysis tool set for population genomics. Mol. Ecol..

[45] Bolger A.M., Lohse M., Usadel B. (2014). Trimmomatic: A flexible trimmer for Illumina sequence data. Bioinformatics.

[46] Marinček P., Pittet L., Wagner N.D., Hörandl E. (2023). Evolution of a hybrid zone of two willow species (*Salix* L.) in the European Alps analyzed by RAD-seq and morphometrics. Ecol. Evol..

[47] Langmead B., Salzberg S.L. (2012). Fast gapped-read alignment with Bowtie 2. Nat. Methods.

[48] Mastretta-Yanes A., Arrigo N., Alvarez N., Jorgensen T.H., Piñero D., Emerson B.C. (2015). Restriction site-associated DNA sequencing, genotyping error estimation and de novo assembly optimization for population genetic inference. Mol. Ecol. Resour..

[49] Rochette N.C., Catchen J.M. (2017). Deriving genotypes from RAD-seq short-read data using Stacks. Nat. Protoc..

[50] Alexander D.H., Novembre J., Lange K. (2009). Fast model-based estimation of ancestry in unrelated individuals. Genome Res..

[51] R Core Team (2012). R: A Language and Environment for Statistical Computing.

[52] Oksanen J., Kindt R., Legendre P., O’Hara B., Stevens M.H.H., Oksanen M.J., Suggests M.A.S.S. (2009). The vegan package. Community Ecol. Package.

[53] Stamatakis A. (2014). RAxML version 8: A tool for phylogenetic analysis and post-analysis of large phylogenies. Bioinformatics.

[54] Excoffier L., Smouse P.E., Quattro J.M. (1992). Analysis of molecular variance inferred from metric distances among DNA haplotypes: Application to human mitochondrial DNA restriction data. Genetics.

[55] Chen H., Boutros P.C. (2011). VennDiagram: A package for the generation of highly-customizable Venn and Euler diagrams in R. BMC Bioinform..

[56] Posada D., Crandall K.A., Templeton A.R. (2000). GeoDis: A program for the cladistic nested analysis of the geographical distribution of genetic haplotypes. Mol. Ecol..

[57] Pickrell J.K., Pritchard J.K. (2012). Inference of population splits and mixtures from genome-wide allele frequency data. PLoS Genet..

